# Sequential Surgical Management of a Recurrent Complex Transsphincteric Anal Fistula With Sphincter Disruption: A Case Report

**DOI:** 10.7759/cureus.88123

**Published:** 2025-07-16

**Authors:** Diego Pérez-Valdez, Alfredo Sinahi Abarca-Magallón, Samuel Hernández-Alvarado, Daniel Castañeda-Rodríguez, Daniel Alejandro Valdivieso-Siguenza

**Affiliations:** 1 General Surgery, Hospital General Regional No. 1 “Ignacio García Téllez”, Instituto Mexicano del Seguro Social (IMSS), Mérida, MEX; 2 Colon and Rectal Surgery, Hospital Regional “Lic. Adolfo López Mateos”, Instituto de Seguridad y Servicios Sociales de los Trabajadores del Estado (ISSSTE), Mexico City, MEX

**Keywords:** anal fistula, colorectal surgery, complex anal fistula, fibrin sealant, ileostomy, sphincteroplasty, stepwise surgical management

## Abstract

High transsphincteric anal fistulas represent a significant therapeutic challenge due to the risk of fecal incontinence associated with division of the sphincter complex. Management must be individualized, particularly in recurrent cases. We report the case of a 55-year-old male with a recurrent high posterior transsphincteric anal fistula who had undergone multiple previous surgical interventions. Initial treatment with fistulectomies and cryptectomies resulted in disruption of both the internal and external anal sphincters, leading to severe fecal incontinence. Due to persistent fistulous drainage, the patient underwent diagnostic laparoscopy with creation of a protective loop ileostomy, irrigation and drainage of the tract, primary transanal closure of the internal opening, and application of fibrin sealant (Tisseel^®^, Baxter Healthcare Corporation, Deerfield, IL). In the outpatient setting, tract curettage using a CitoBrush^®^ (Medscand AB, Malmö, Sweden) and repeat application of fibrin sealant were performed. Subsequent examination under anesthesia revealed fibrosis without evidence of an active tract. Intestinal continuity was ultimately restored. The patient’s postoperative course was favorable, with no recurrence or continence impairment. This case illustrates the importance of a stepwise surgical approach integrating conventional and adjunctive techniques for the treatment of complex anal fistulas. The combined use of sphincteroplasty, temporary fecal diversion, and biological sealants provided effective disease control while preserving sphincter function. Current evidence supports the use of fibrin sealants as adjuncts in fibrotic tracts and the role of temporary diversion in cases involving sphincter disruption. Sequential and patient-specific surgical management led to successful resolution of the fistula without compromising continence. This multidisciplinary strategy may be considered in patients with complex anatomy and a history of multiple surgical failures.

## Introduction

High transsphincteric anal fistulas are classified as complex due to their anatomical characteristics and the risk of fecal incontinence associated with sphincter division. These fistulas are particularly challenging in recurrent cases or when associated with suprasphincteric extensions, requiring individualized treatment strategies that balance eradication of the fistula with preservation of sphincter function [1,2].

Several treatment options have been described for transsphincteric fistulas, including fistulotomy, loose or cutting seton placement, endorectal advancement flaps, and ligation of the intersphincteric fistula tract (LIFT). While these techniques may be effective in selected cases, no single approach has demonstrated consistent superiority across all presentations. Complex or recurrent fistulas often require a multimodal and staged approach, tailored to the patient’s specific anatomy and surgical history [3,4].

Among the available surgical techniques for managing transsphincteric fistulas, options such as cutting setons, LIFT, and video-assisted anal fistula treatment (VAFT) have been described with varying degrees of success. These methods may be suitable in select patients with favorable anatomy and limited sphincter involvement. However, in cases of high posterior tracts or when significant sphincter disruption is present, as in the case reported here, these approaches may not be feasible or effective, necessitating a more individualized and reconstructive strategy.

Magnetic resonance imaging (MRI) is the current gold standard for preoperative assessment, offering detailed visualization of fistulous tracts, secondary extensions, internal openings, and their relationship to the sphincter complex. It also plays an emerging role in postoperative monitoring and early detection of recurrence. Recent advances, such as MRI-based predictive nomograms incorporating quantitative metrics like contrast-to-noise ratio (CNR) and apparent diffusion coefficient (ADC), have shown promise in assessing treatment response and guiding decision-making [1].

Despite improvements in imaging and surgical techniques, recurrence rates remain substantial. In cases of iatrogenic sphincter disruption, reconstructive strategies such as sphincteroplasty may be indicated to restore function [2,5]. Adjunctive therapies, including fibrin sealants, have also been explored with encouraging results, particularly in fibrotic or blind-ending tracts and when used in conjunction with diversion or immunomodulation [2,4]. Temporary fecal diversion may facilitate healing in selected patients by reducing local contamination and inflammation, especially in the context of complex anatomy or failed prior surgeries [6,7].

This report describes the case of a 55-year-old male with a recurrent high posterior transsphincteric anal fistula and complete midline disruption of both sphincters, successfully managed through a stepwise, image-guided surgical strategy. The combination of advanced imaging, targeted sphincteroplasty, temporary fecal diversion, and fibrin sealant allowed for durable fistula resolution while preserving continence.

## Case presentation

A 55-year-old male from Chiapas, residing in Mexico City, with a history of hypothyroidism managed with levothyroxine and occasional alcohol use, presented in September 2021 with a right gluteal abscess. After failed outpatient management, bedside surgical drainage was performed. Despite initial improvement, the patient evolved into a complex anal fistula.

Colorectal surgery evaluation confirmed a posterior transsphincteric fistula of cryptoglandular origin. In August 2022, he underwent fistulectomy, internal opening closure, and cryptectomy. However, recurrence was documented during follow-up, with a new external opening in the posterior right gluteal region, approximately 9 cm from the anal verge.

In July 2023, a second surgical intervention included fistulectomy, cryptectomy, sphincterotomy, and sphincteroplasty of both the internal and external anal sphincters. Postoperatively, the patient developed severe fecal incontinence due to midline posterior disruption of both sphincters, in addition to fistula recurrence.

**Figure 1:**
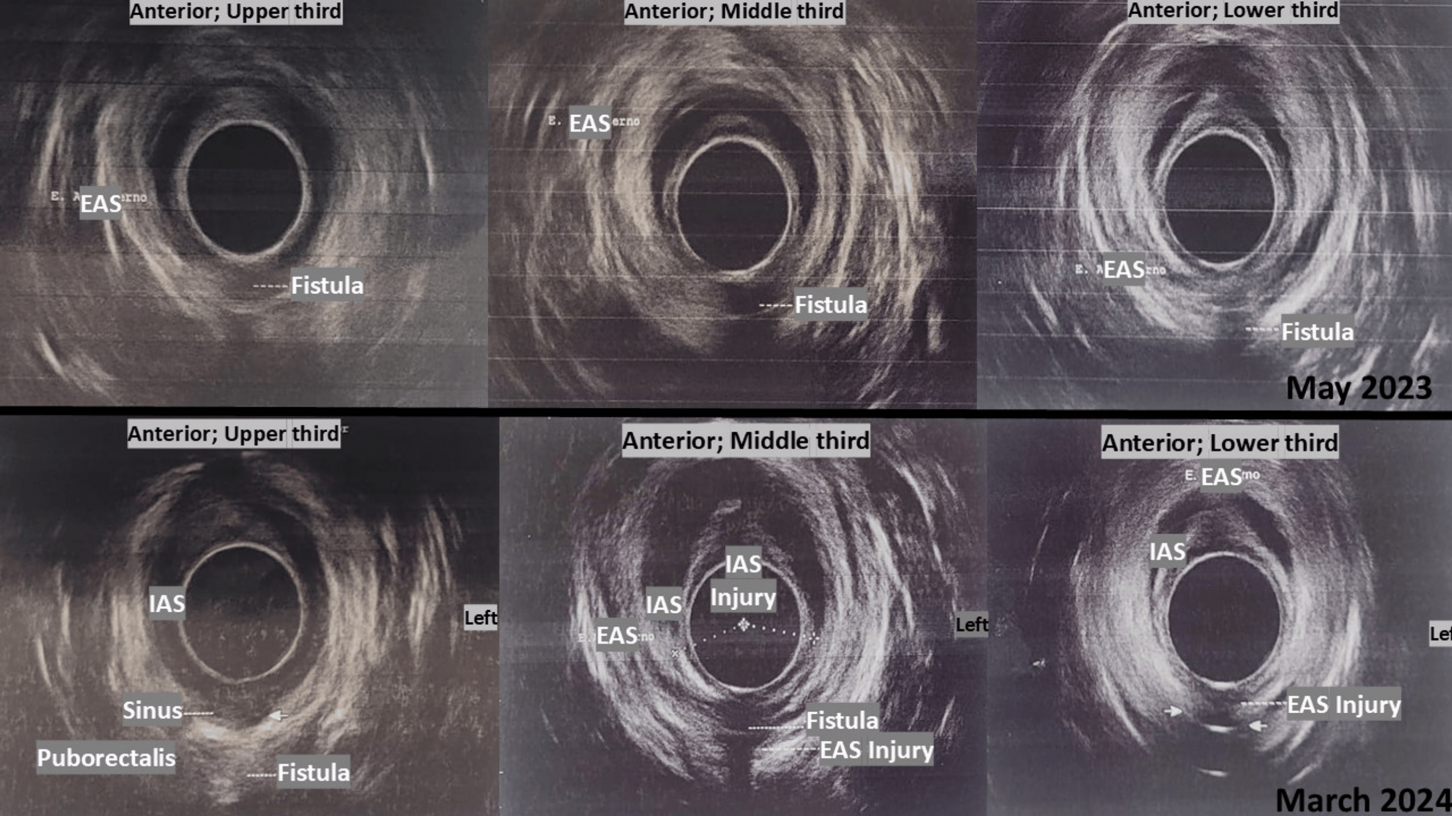
Endoanal ultrasound findings (May 2023 and March 2024) 360° endoanal ultrasound images showing the progression of a complex posterior transsphincteric fistula. In May 2023 (top row), a hypoechoic fistulous tract is visualized. In March 2024 (bottom row), recurrence with a fibrotic sinus tract extending toward the puborectalis muscle is observed, along with clear disruption of both the internal and external anal sphincters. EAS, external anal sphincter; IAS, internal anal sphincter.

A 360° endoanal ultrasound performed in May 2023 confirmed the presence of a complex posterior fistula (Figure 1, top row). A follow-up study in March 2024 revealed recurrence with a fibrotic sinus tract extending toward the puborectalis muscle and evident sphincter disruption (Figure 1, bottom row).

**Figure 2:**
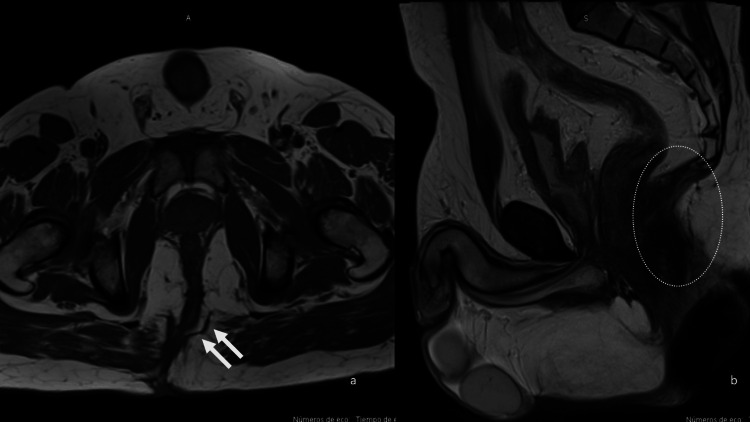
Pelvic MRI showing a recurrent high posterior transsphincteric fistula (June 2024) Axial T2-weighted image demonstrating a hyperintense tract traversing the external and internal anal sphincters, consistent with a high posterior transsphincteric fistula; see arrows (a). Sagittal view revealing extension of the fistulous tract toward the puborectalis muscle, with associated sphincter complex discontinuity and perianal fat infiltration, see circle (b). MRI, magnetic resonance imaging.

**Figure 3:**
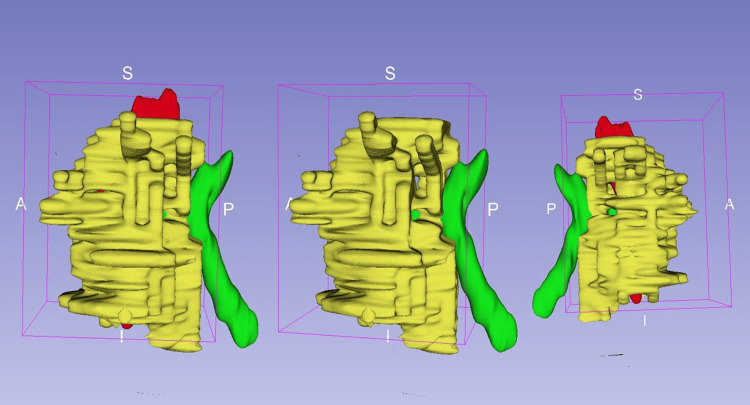
Three-dimensional reconstruction of the complex posterior transsphincteric anal fistula A three-dimensional digital model generated from pelvic MRI segmentation, used for surgical planning. The external anal sphincter is shown in yellow, the internal anal sphincter in red, and the fistulous tract in green. This visualization aided in understanding the spatial relationship between the fistulous pathway and the sphincter complex, guiding a tailored surgical approach. Axes: A (anterior), P (posterior), S (superior), I (inferior). MRI, magnetic resonance imaging.

In June 2024, pelvic MRI identified a high posterior transsphincteric fistula with a blind-ending tract and confirmed the disruption of the sphincter complex (Figure 2). A three-dimensional (3D) reconstruction was generated from the MRI data to assist in spatial orientation and preoperative planning. This model depicted the anatomical relationship between the fistulous tract and the sphincter apparatus (Figure 3). A posterior fistulectomy with sphincteroplasty was performed. 

Three weeks later, persistent symptoms prompted a diagnostic laparoscopy and creation of a protective loop ileostomy. Examination under anesthesia revealed a posterior transsphincteric fistula with a 1 × 1 cm internal rectal opening, containing food particles, purulent discharge, and debris. The blind tract extended toward the puborectalis sling and originated from a midline posterior crypt. Transanal irrigation and drainage were carried out, followed by primary internal opening closure and fibrin sealant (Tisseel^®^, Baxter Healthcare Corporation, Deerfield, IL) application.

Due to delayed healing, outpatient tract curettage using a CitoBrush^®^ (Medscand AB, Malmö, Sweden) and repeat fibrin sealant application were performed in September 2024.

In February 2025, a follow-up examination under anesthesia demonstrated fibrotic tissue in the posterior anal canal and mid-rectum, with no active fistulous tract. Intestinal continuity was restored in April 2025. The patient’s postoperative course was favorable, with no evidence of recurrence or fecal incontinence. The complete timeline of procedures is presented in Table 1.

**Table 1:** Timeline of surgical procedures for recurrent complex anal fistula

Date	Procedure
08/08/2022	Fistulectomy, fistulous tract excision (cut-out), closure of primary orifice, and cryptectomy
03/07/2023	Fistulectomy, cryptectomy, sphincterotomy, and sphincteroplasty (internal and external anal sphincters)
21/06/2024	Posterior fistulectomy and sphincteroplasty due to midline posterior sphincter discontinuity
15/07/2024	Diagnostic laparoscopy, protective loop ileostomy, fistula irrigation and drainage, primary transanal closure, and fibrinogen sealant (Tisseel®)
24/07/2024	Exploratory laparotomy for bowel obstruction, end ileostomy, and management of sphincteroplasty dehiscence
04/09/2024	Office-based curettage using CitoBrush^®^ and reapplication of fibrin sealant (Tisseel®)
07/04/2025	Restoration of intestinal continuity

## Discussion

The management of recurrent complex transsphincteric anal fistulas remains a significant surgical challenge. These fistulas are commonly associated with prior treatment failure, anatomical distortion, and a high risk of sphincter dysfunction. In our case, multiple unsuccessful interventions led to disruption of both sphincters, requiring a patient-specific, staged approach that prioritized infection control and continence preservation.

MRI played a central role in surgical planning, allowing detailed assessment of the fistulous tract, fibrosis, and sphincter involvement. Advanced imaging-based predictive tools have demonstrated value in monitoring healing and guiding follow-up strategies. Xu et al. validated an MRI-based nomogram for early outcome prediction, and Emile et al. emphasized the utility of imaging in assessing recurrence risk. In our case, the use of 3D reconstruction further enhanced spatial orientation in a surgically altered anatomical field [1,6].

In addition to MRI, 360° 3D endoanal ultrasound was employed during both initial and follow-up evaluations. This modality enabled real-time assessment of the fistulous tract, its relationship with sphincter structures, and the presence of fibrotic changes. While MRI remains the gold standard, 3D endoanal ultrasound offers complementary diagnostic value, particularly in postoperative settings or when MRI access is limited.

Fistula recurrence is influenced by multiple factors, including tract height, previous surgeries, missed extensions, and failure to identify the internal opening. Ng et al. identified suprasphincteric tracts, female sex, and prior interventions as independent predictors of poor healing [8]. In our patient, these elements were central to surgical decision-making.

Sphincteroplasty was indicated due to documented disruption of both the internal and external anal sphincters. Although not routinely used in cryptoglandular fistulas, selected reports support its use in cases of focal, midline defects. Readi et al. demonstrated functional recovery in similar scenarios [5].

Temporary fecal diversion facilitated local control of sepsis and healing of the repaired sphincters. Although traditionally reserved for Crohn’s disease or severe perianal infection, recent literature supports its selective use in complex cryptoglandular fistulas. Sørensen et al. [9] and Abbas et al. [7] reported improved healing with diversion in high-risk cases, and Mei et al. [10] emphasized the importance of delaying stoma closure until full clinical and radiologic resolution.

Adjunctive fibrin sealant was employed in the final stage. Although outcomes vary, Vidon et al. [3] showed clinical remission at one year in patients with Crohn’s-related fistulas, particularly when used alongside immunosuppression. Litta et al. [11] also reported success in cryptoglandular tracts with limited fibrosis. In contrast, platelet-rich fibrin (PRF) as described by Pérez Lara et al. may be suitable for low-complexity tracts but is not indicated in patients with sphincter disruption [4].

This case contributes to the existing literature by documenting a multidisciplinary, image-guided approach for managing a rare presentation of a recurrent high posterior transsphincteric anal fistula with bilateral sphincter disruption. It illustrates how 3D endoanal ultrasound, MRI-based surgical planning, midline sphincteroplasty, temporary diversion, and biological sealants can be integrated into a structured algorithm to achieve continence-preserving resolution in surgically complex scenarios [12].

This case report was prepared in accordance with the SCARE (Surgical CAse REport) guidelines to ensure transparency and completeness of reporting [13].

## Conclusions

Recurrent high transsphincteric anal fistulas represent a significant surgical challenge, especially when previous interventions have resulted in anatomical distortion or sphincter disruption. This case demonstrates that a tailored, stepwise approach, guided by high-resolution imaging and incorporating reconstructive techniques, temporary fecal diversion, and adjunctive fibrin sealant, can achieve durable fistula resolution while preserving continence in anatomically compromised patients.

MRI and 3D modeling were instrumental in surgical planning, enabling accurate delineation of the fistulous tract and associated damage. Sphincteroplasty allowed for anatomical and functional reconstruction, while temporary fecal diversion created optimal local conditions for healing. The application of fibrin glue, following adequate drainage and tract conditioning, served as an effective adjunct to complete the closure process without additional invasive procedures.

Beyond clinical success, this case contributes to the existing literature by illustrating the feasibility of integrating advanced imaging, reconstructive surgery, fecal diversion, and biological sealants in the management of complex fistulas with sphincter disruption. The structured, reproducible strategy outlined here offers practical guidance for surgeons facing similar high-risk cases, emphasizing the importance of individualized, multimodal treatment in preserving function and achieving long-term resolution.
